# The System of Clinical Instruction in the Italian Schools

**Published:** 1848-01

**Authors:** 


*The system of Clinical Instruction in the Italian Schools.*—"The hospitals generally are large, containing from one to three thousand beds, and there are four clinical wards for the male and female medical and surgical cases. Each of these is under the direction of a clinical professor, who takes his turn of duty for a year or session. The students are obliged to attend punctually at the morning and evening visits. Each patient is consigned to one of the students, who, before the visit, obtains from the patient or his friends, his name, country, condition, age, progress of the disease, stage of the disease, exciting causes, treatment that has been adopted, and present symptoms. This is all written upon paper ruled for the purpose, and when the professor or his assistant visits the patient, the student reads it, and explains the reason of his diagnosis. Afterwards, every morning and evening he registers the most important changes in symptoms and treatment. Before the patient leaves the hospital, the student reads his account of the case to the professor, and it is then deposited in the records of the clinical ward. If the patient dies, before the body is opened the student reads the history of the case, with the diagnosis and remarks of the professor; and then, other students examining the various organs, he writes an exact account of what is observed, and remarks upon the points in confirmation of, or in opposition to, the diagnosis made during life. If any student refuses to attend the patient consigned to him, or does not keep the history of the case, and consign it to the clinical archives, he is not admitted to scholastic examination; while assiduity and exactness are favourably considered at his examinations, and in the distribution of honours and profit."—*British and Foreign Medical Review.*

